# Molecular Detection of Parvovirus in Captive Siberian Tigers and Lions in Northeastern China From 2019 to 2021

**DOI:** 10.3389/fmicb.2022.898184

**Published:** 2022-05-12

**Authors:** Shuping Huang, Xiang Li, Wei Xie, Lijun Guo, Dan You, Haitao Xu, Dan Liu, Yulong Wang, Zhijun Hou, Xiangwei Zeng, Siyuan Yang, Hongliang Chai, Yajun Wang

**Affiliations:** ^1^College of Wildlife and Protected Area, Northeast Forestry University, Harbin, China; ^2^Siberian Tiger Park, Harbin, China; ^3^Heilongjiang Vocational College for Nationalities, Harbin, China

**Keywords:** Siberian tigers, lion, FPV, VP2 gene, nested PCR, real-time PCR

## Abstract

The fact that wild felines are carriers of pernicious infectious viruses should be a major concern due to the potential cross-species transmission between the felines and human or domestic animals. However, studies on the virus in the captive wild felines, especially in tigers, are thin on the ground. In this study, we screened four infectious viruses, namely, feline parvovirus (FPV), feline coronavirus (FCoV), canine distemper virus (CDV), and influenza A virus (IAV), in the blood samples of 285 captive Siberian tigers (*Panthera tigris altaica*) and in the spleen samples of two deceased lions (*Panthera leo*), which were collected from 2019 to 2021 in three Siberian Tiger Parks from the northeast of China. Nucleic acids isolated from the blood samples collected from tigers and the spleen samples collected from two deceased lions were positive for FPV by PCR, and the positive rate was 4.6% (13/285) in tigers. Furthermore, the VP2 gene of FPV was amplified by nested PCR, and the sequences of the VP2 gene from these six FPV positive strains shared 98.3–99.9% homology with the reference. The key amino acid sites of VP2 protein were consistent with that of FPV reference strains. Phylogenetic analysis based on the VP2 gene showed that in this study, FPV-positive strains were grouped within the FPV clade and closely related to the Asian strains clade. The results of this study showed that FPV circulated in the captive Siberian tigers and lions in northeastern China and provided valuable information for the study of FPV epidemiology in wild felines. Therefore, we suggest that regular antibody monitoring and booster immunization for tigers should be performed.

## Introduction

The captive breeding mode of feline species increases the possibility of cross-species transmission between the species and humans (Foley et al., 2013; Carver et al., 2016), which can potentially impact the survival of wildlife populations. For example, feline parvovirus (FPV), feline coronavirus (FCoV), canine distemper virus (CDV), and influenza A virus (IAV) are widespread in wild felines (Duarte et al., 2012; Candela et al., 2019; McCauley et al., 2021). FPV is a pathogen that causes panleukopenia in felines, the symptoms of which include high fever, vomiting, severe leukopenia, and enteritis (Stuetzer and Hartmann, 2014). It is closely related to blue fox parvovirus (BFPV), mink enteritis virus (MEV), raccoon dog parvovirus (RDPV), and canine parvovirus (CPV) in gene, structure, and antigen. FPV affects cats of all ages and has a wide range of hosts, affecting all members of the family felines (Allison et al., 2013). Goodrich et al. (2012) found that the antibody-positive rate of FPV among Siberian tigers in eastern Russia was 68% (*n* = 44). In addition, FPV exposure or infection has been reported in captive Siberian tigers in Guangzhou and Jilin, China (Wang et al., 2017, 2019). CDV is a multi-host pathogen with various clinical outcomes of interspecies and cross-species transmission. PCR and antibody detection of CDV infection have been reported in wolves, tigers, lions, ferrets, minks, red foxes, giant pandas, and genets (Feng et al., 2016; Loots et al., 2017; Weckworth et al., 2020). FCoV infects domestic cats and wild felines; although FCoV tends to cause mild or inconspicuous illness, a small percentage of felines die from the fatal systemic disease feline infectious peritonitis (FIP) (Stout et al., 2021). IAV infects a variety of vertebrates, including birds, humans, and other mammals (Driskell et al., 2013). Although felines were previously considered to be resistant to IAV infection, the H5N1 subtype virus is wreaking havoc in many countries and has caused fatal infection in domestic cats, leopards, and tigers (Driskell et al., 2013; He et al., 2015).

To investigate the prevalence of FPV, FCoV, CDV, and IAV in Siberian tigers and lions in northeastern China, and to enrich the epidemiological data of tigers, FPV, FCoV, CDV, and IAV were detected by PCR, and the key amino acid site mutation and phylogenetic analysis of VP2 gene were performed.

## Materials and Methods

### Samples Collection

A total of 285 blood samples of captive Siberian tigers were randomly collected from the Siberian Tiger Parks in three cities: Harbin (*n* = 190, 200–300 tiger/km^2^), Hailin (*n* = 55, 1,300–1,400 tiger/km^2^), and Shenyang (*n* = 40, 600–700 tiger/km^2^) in northeastern China (41–46°N, 122–130°E), from January 2019 to March 2021. The samples used in this study are the same as our previous studies, but after the detection of four viruses [i.e., feline herpesvirus 1 (FHV-1), feline calicivirus (FCV), feline immunodeficiency virus (FIV), and feline leukemia virus (FeLV)] and persistent organic pollutants, some samples have been run out, so the sample size in this study is 285, smaller than the sample size of 324 last time (Liu et al., 2021a,b; Huang et al., 2022). All tigers fasted for 1 day before the blood sample collection, and the tigers were anesthetized with a dart containing 10 mg/kg of ketamine (Jiangsu Zhongmu Beikang Pharmaceutical Co., Ltd., China). In addition, spleen samples were collected from two dead lions in the Siberian Tiger Park of Harbin, and all samples were stored at −80°C for later use. All tigers were dewormed once a year (Hailechong, Zhejiang Hai Zheng Animal Health Products Co., Ltd., China) and were vaccinated twice. The first immunization was taken after birth and the second was 1 month later (Fel-O-Vax PCT, Zoetis, United States). No animals were killed for investigative purposes, and all samples were collected by the veterinarian of Siberian Tiger Park, which was approved by the Northeast Forestry University Institutional Review Board of Ethics and Administration of Experimental Animals (NEFU-IRBEA) (Liu et al., 2022).

### Virus Detection by PCR

The viral nucleotide extraction from the blood of tigers and the spleen of deceased lions was performed by the Baypure™ Universal Magnetic Bead Viral DNA/RNA Rapid Extraction Kit (BayBio, Guangzhou, China), and reverse transcription was performed to get the cDNA of FCoV. The extracted DNA, RNA, and cDNA were stored at −80^°^C.

Feline parvovirus, CDV, and IAV were detected by real-time PCR, and FCoV was detected by nested PCR, as previously described (Herrewegh et al., 1995; Meli et al., 2009; Streck et al., 2013); the PCR primers are shown in Supplementary Table 1. VP2 gene of FPV was amplified by nested PCR (Mochizuki et al., 1996; Steinel et al., 2000; Battilani et al., 2001; Sacristán et al., 2021). FPV and FCoV positive controls were cat samples with the corresponding disease, which were collected in pet hospitals. CDV positive control is the CDV live attenuated vaccine. Birds infected with IAV are used as positive controls for testing IAV. To avoid contamination of samples, RNase-free water was used as a negative control for each assay.

### Phylogenetic Analysis

Multiple sequence alignments were conducted using the MegAlign program of DNAStar 11 software and were translated into putative amino acid sequences using the MEGA 7.0 software.^1^ Reference sequences were selected based on the VP2 gene of panleukopenia viruses from the GenBank database.^2^ The phylogenetic tree was established in MEGA 7.0 software. The neighbor-joining method with 1,000 bootstrap replications was used with the Kimura 2-parameter model to analyze the relationship between the partial VP2 sequences in this study and other reference sequences.

## Results

In this study, only parvovirus infection was detected in the tigers (13/285; 4.6%) and two deceased lions (2/2; 100%) from Harbin, but no FCoV-, CDV-, and IAV-positive samples were found. FPV-positive samples were detected only in the Harbin area, and there is no much difference in the positive rate between male (4.9%) and female (4.3%) animals. However, the positive rate of young tigers (5.5%) was higher than that of adult tigers (3.8%) (Table 1).

**TABLE 1:** Information of FPV positive samples, including the number and positive rate by species, sex, age, and region.

Species	Sex/positive samples (rate)	Age/positive samples (rate)	Region/positive samples (rate)
Siberian tiger (*Panthera tigris altaica)* (*n* = 285)	male (*n* = 144)/7 (4.9%) female (*n* = 141)/6 (4.3%)	young (*n* = 128)/7 (5.5%) adult (*n* = 157)/6 (3.8%)	Harbin (*n* = 190)/13 (6.8%) Hailin (*n* = 55)/0
			Shenyang (*n* = 40)/0
Lion (*Panthera leo*)	male (*n* = 1)/1 (100%)	young (*n* = 1)/1 (100%)	Harbin (*n* = 2)/2 (100%)
(*n* = 2)	female (*n* = 1)/1 (100%)	adult (*n* = 1)/1 (100%)	

In this study, we have got six partial VP2 gene sequences and approximately 800 bp fragments from five positive strains (i.e., HB1807, HB1752, HB1765, HB1819, and LWL) and 1,683 bp from the HB1811 strain. Multiple sequences alignment based on the nucleotide (nt) of the VP2 gene showed that six positive strains shared 99.3–99.9% homology with FPV isolates and 98.3–98.9% homology with CPV isolates. The amino acids at 80, 93, 103, 323, 564, and 568 of the VP2 gene were the key sites to distinguish FPV and CPV antigen types (Yi et al., 2021). To further analyze the mutation of positive strains, the amino acid sequences of VP2 are compared in Table 2. The result showed that the amino acid mutation sites of six VP2 proteins were identical with FPV reference strains. However, due to the quality of samples or primers, the full-length sequence of the VP2 gene was not successfully amplified.

**TABLE 2:** Amino acid residues characteristic and pairwise identity of VP2 in this study compared with other related parvovirus strains.

Strain	The main amino acid sites												Nucleotide identity (%)	Antigenic type
	80	87	93	101	103	297	300	305	323	426	564	568		
HB1807*	K	M	K	T	V	S	A	D	D	N	–	–	99.3–100%	TPV
HB1752*	–	–	–	–	–	–	–	D	D	N	N	–		TPV
HB1765*	–	–	–	–	–	S	A	D	D	N	N	–		TPV
HB1819*	K	M	K	T	V	S	A	D	D	–	–	–		TPV
HB1811*	K	M	K	T	V	S	A	D	D	N	–	–		TPV
LWL*	–	–	–	–	–	–	–	–	D	N	N	A		FPV
AB054227	K	M	K	T	V	S	A	D	D	N	N	A	99.4–99.9%	TPV
FJ405225	K	M	K	T	V	S	A	D	D	N	N	A	99.3–99.6%	TPV
EU697384	K	M	K	T	V	S	A	D	D	N	N	A	99.3–99.6%	TPV
M38246	K	M	K	I	V	S	A	D	D	N	N	A	99.4–99.6%	FPV
EU498680^#^	K	M	K	I	V	S	A	D	D	N	N	A	99.3–99.6%	FPV
EU498681^#^	K	M	K	T	V	S	A	D	D	N	N	A	99.3–99.6%	FPV
M38245	R	M	N	I	A	S	A	D	N	N	S	G	98.9–99.9%	CPV-2
M24003	R	L	N	T	A	S	G	Y	N	D	S	G	98.7–98.9%	CPV-2a
AF306444	R	L	N	T	A	A	G	Y	N	D	S	G	98.3–98.9%	CPV-2b
KP682519	R	L	N	T	A	A	G	Y	N	E	S	G	98.4–98.7%	CPV-2c

“–” represents sequencing failure; “*” represents the positive strains in this study. The first five samples are tiger samples, and LWL sample is lion sample. “^#^” represents vaccine strains. EU498680: Purevax Merial vaccine, EU498681: Felocell Pfizer vaccine.

**FIGURE 1:**
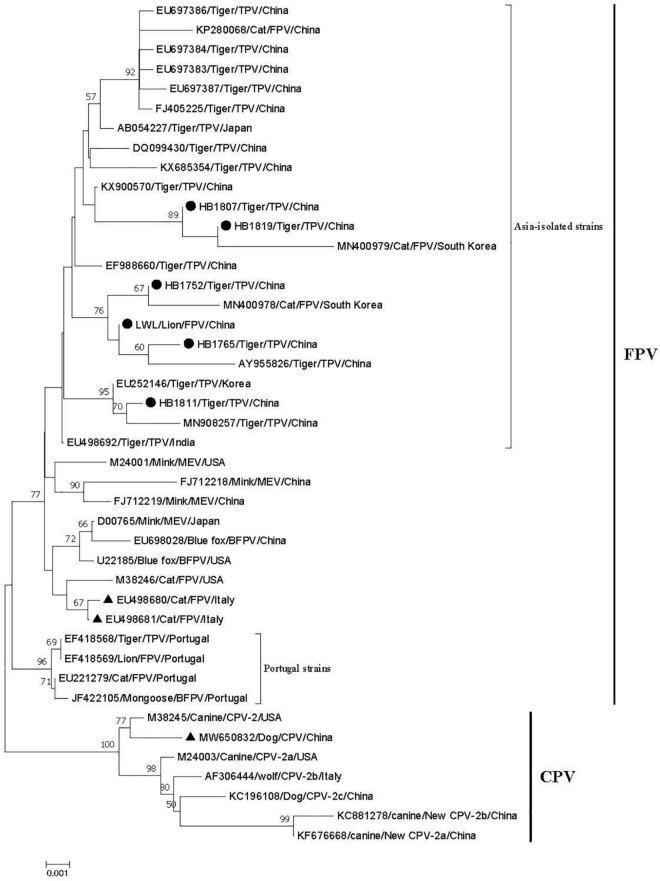
Phylogenetic tree of six FPV partial VP2 genes compared with other references of parvovirus strains obtained from GenBank. The tree was generated using the neighbor-joining method in MEGA 7.0 using the Kimura 2-parameter model with 1,000 bootstrap values. Bootstrap values ≥ 50 at the nodes of the tree. “●” represents the strains in this study; “▲” represents vaccine strains; EU498680 and EU498681 are the FPV vaccine strain; MW650832 is the CPV vaccine strain. The length of the six VP2 genes is as follows: HB1807: 973 bp; HB1752: 790 bp; HB1765: 984 bp; HB1819 bp: 973; HB1811: 1683 bp; LWL: 825 bp.

A phylogenetic tree about the VP2 nucleotide sequences was constructed based on the positive strain sequences in this study and 37 reference sequences from Europe, America, and Asia (Supplementary Table 2). The phylogenetic analysis indicated that all the parvoviruses can be grouped into two large branches: the FPV branch and the CPV branch (Figure 1). FPV branch comprised TPV (The feline parvovirus isolated from tigers was named TPV), FPV, MEV, and BFPV. In the FPV branch, 26 strains from China, Korea, Japan, India, United States, and Italy formed a cluster, and four sequences from Portugal (EF418568, EF418569, EU221279, JF422105) formed a different clade. All positive strains in this study belong to the FPV strains branch and were closely related to the Asia-isolated strains, but they were distant from the Portugal-isolated strains. The Asian isolates were mainly divided into two differentiated clades, and HB1811 strain was closely related to a tiger strain isolated in China (MN908257), but was not in the same clade as other strains in this study.

## Discussion

Wild felines are susceptible to virus infection due to cross-species transmission potentially by domestic cats or dogs (Canuti et al., 2020). Early detection of viral shedding in asymptomatic or symptomatic felines may help inspect the health status of wild felines and are critical for early treatment and prevention plans for animal diseases. In this study, the positive rate of FPV in captive Siberian tigers was 4.6%, higher than previous results of FPV in Malaysian tigers was 2.7% (Nur-Farahiyah et al., 2021), and the positive rate of the virus in Italian red foxes was 2.8% (Ndiana et al., 2021). The tigers in this study are captive, and such a large sample size may be the main reason for this result. Furthermore, there are no coinfection among FPV and the other four viruses (i.e., FHV-1, FCV, FIV, and FeLV) (Huang et al., 2022). Real-time PCR detection of FPV in the two dead deceased lions was also positive. The two deceased lions showed depressed spirit, lost appetite with shame and tears, dry nose, dark red vomit, bloody stool, dehydration, a body temperature of 39.5°C, and leukocyte elevation during the paroxysm of symptoms. The pathological changes after autopsy mainly included gastric bleeding, bile reflux, severe intestinal bleeding, hemorrhagic enteritis, mesenteric lymphadenitis enlargement, and hemorrhage, so we believe that FPV infection may be the main reason for the death of lions. These results suggest that FPV has a certain prevalence in captive Siberian tigers and lions in northeastern China. However, we have not found FCoV, CDV, and IAV in any of these samples, which showed that there was no epidemic of FCoV, CDV, and IAV in the three Siberian Tiger parks.

Feline parvovirus was first discovered in 1928, and canine parvovirus type 2 (CPV-2) was discovered in 1978. CPV-2 originated from FPV or an FPV-like parvovirus in carnivores. In 1979, CPV-2a mutated away from the original type 2 in five amino acids (aa) in VP2 that alter relevant biological properties between the strains. Another antigenic variant, CPV-2b, with a single additional substitution (Asn426Asp) in the VP2 protein appeared in 1984 and spread globally (Parrish et al., 1985; Parrish, 1991). Since then, many new CPV variants throughout the world have been detected, such as the “new CPV-2a” and “new CPV-2b” with an additional 297-Ala mutation, CPV-2c with an additional 300-Asp mutation, and 426-Glu mutation (Buonavoglia et al., 2001; Decaro and Buonavoglia, 2012). In this study, due to the sample’s quality and DNA concentration reasons, such as samples stored for a long time, repeated freezing, and thawing, we only got six positive sample sequences, and we failed to amplify the entire VP2 gene. The main amino acid mutation sites obtained in this study were identical to FPV (Table 2).

The viruses are named after the host from which they are isolated, such as FPV, TPV, MEV, BFPV, and CPV. In this study, five TPV strains from tigers and one FPV (LWL) of lion strain are identified. We selected 37 parvovirus VP2 genes from GenBank for phylogenetic analysis, and all six positive strains in this study belong to the Asia-isolated FPV strains but had a distant relationship to the Portugal-isolated FPV strains. These results suggest that the spreading and prevalence of FPV are related to the geographical location of the animals. The relationship between positive strains and commercial FPV vaccine strains (i.e., EU498680 and EU498681) is also distant. Inactivated vaccines (Fel-O-Vax PCT, Zoetis, United States), which combine FCV, FHV, and FPV strains, are used to prevent disease in tigers. However, tigers and lions are still infected with FPV; the reason for this result may be the low antibody level and short antibody duration of the FPV-inactivated vaccine (Bergmann et al., 2019). In the FPV clade, both the tigers and lion strains in this study belong to the same evolutionary clade, and in this clade, the tiger and lion strains are closely related to the reference strains from tigers and cats. The Siberian tigers in these three regions were all fenced with wire mesh that prevents the tigers from escaping but does not restrict domestic cat movement, and the staffs often see the stray cats pass in and out of the Siberian Tiger Park freely, which allows for the possibility of contact, and therefore transmission, between the stray cats and tigers or lions (Liu et al., 2022). Considering the widespread infection of FPV (Franzo et al., 2017) and the captive environment, FPV might be transmitted from the stray cats to tigers and lions. Previous studies have confirmed the possibility of cross-species transmission of FPV in domestic cats and other felines (Wang et al., 2017; Shetty et al., 2019). Furthermore, human participation in the landscape is an important factor affecting the spread of pathogens across species (Carver et al., 2016).

Combined with our previous study (Huang et al., 2022), FHV-1, FCV, FIV, and FPV have certain prevalence rates in the Siberian Tiger Park of northeastern China, and interspecies and cross-species transmission may happen. This study not only provides new data on the prevalence of FPV among tigers and lions but also contributes to improving the management of captive felines.

## Data Availability Statement

The data presented in the study are deposited in the NCBI repository, accession numbers OM810192, OM810193, OM810194, OM810195, and OM810196.

## Ethics Statement

The authors confirm that the ethical policy has been followed as noted on the author guidelines page of the journal, and all blood samples and swabs of the Siberian tigers were collected by the Siberian Tiger Park, which was approved by the Northeast Forestry University Institutional Review Board of Ethics and Administration of Experimental Animals (NEFU-IRBEA).

## Author Contributions

All authors of this research manuscript have directly participated in the planning, execution, and analysis of the study.

## Conflict of Interest

The authors declare that the research was conducted in the absence of any commercial or financial relationships that could be construed as a potential conflict of interest.

## Publisher’s Note

All claims expressed in this article are solely those of the authors and do not necessarily represent those of their affiliated organizations, or those of the publisher, the editors and the reviewers. Any product that may be evaluated in this article, or claim that may be made by its manufacturer, is not guaranteed or endorsed by the publisher.

## Footnotes

1
http://www.megasoftware.net/

2
https://www.ncbi.nlm.nih.gov/nucleotide/
