# Supplementary material for: Molecular Detection of Parvovirus in Captive Siberian Tigers and Lions in Northeastern China From 2019 to 2021
Source: Front Microbiol. 2022 May 12;13:898184. doi: 10.3389/fmicb.2022.898184 (PMC9133805; doi:10.3389/fmicb.2022.898184)
Supplement: Supplementary file 1 [file Table_1.docx]

**Supplementary Table 1.** Primers of PCR for FPV, FCoV, CDV, and IAV

| Virus | Primer | Sequence (5′ to 3′) | Sense | Reference |
| --- | --- | --- | --- | --- |
| FPV | FPV-F | TGGAACTAGTGGCACACCAA | Forward | (Streck et al., 2013b) |
|  | FPV-R | AAATGGTGGTAAGCCCAATG | Reverse |  |
|  | FPV-P | 6FAM-CAGGTGATGAATTTGCTACAGG-BHQ1 | Probe |  |
| CDV | CDV.78f | GGAAGCCTTGATGATAGCACTGA | Forward | (Meli et al., 2009) |
|  | CDV.161r | GCCGAAAGAATATCCCCAGTT | Reverse |  |
|  | CDVp | 6FAM-TCTGGCGAAGATTATTCCGAAGGAAATGCT-TAMRA | Probe |  |
| IAV | InAM-F | GACCRATCCTGTCACCTCTGAC | Forward |  |
|  | InAM-R | AGGGCATTYTGGACAAAKCGTCTA | Reverse |  |
|  | InAM-P | FAM-TGCAGTCCTCGCTCACTGGGCACG-BHQ1 | Probe |  |
| FCoV | P205-F | GGCAACCCGATGTTTAAAACTGG | Forward | (Herrewegh et al., 1995) |
|  | P211-R | CACTAGATCCAGACGTTAGCTC | Reverse |  |
|  | P276-F | CCGAGGAATTACTGGTCATCGCG | Forward |  |
|  | P204-R | GCTCTTCCATTGTTGGCTCGTC | Reverse |  |
| VP2 gene | VPF | ATGGCACCTCCGGCAAAGA | Forward | (Mochizuki et al., 1996; Steinel et al., 2000; Battilani et al., 2001) |
|  | VPR | TTTCTAGGTGCTAGTTGAG | Reverse |  |
|  | P1 (mc) | ATGAGTGATGGAGCAGTTC | Forward |  |
|  | M5mod | ATAACAAACCTTCTAAATCCTATATCAAAT | Reverse |  |
