# Supplementary material for: Molecular Detection of Parvovirus in Captive Siberian Tigers and Lions in Northeastern China From 2019 to 2021
Source: Front Microbiol. 2022 May 12;13:898184. doi: 10.3389/fmicb.2022.898184 (PMC9133805; doi:10.3389/fmicb.2022.898184)
Supplement: Supplementary file 2 [file Table_2.docx]

**Supplementary Table 2.** Amino acid mutations in VP2 protein of parvovirus

| GenBank number | Animal | Virus type | Country | Mutation sites: amino acid residue | | | | | | | | | | | |
| --- | --- | --- | --- | --- | --- | --- | --- | --- | --- | --- | --- | --- | --- | --- | --- |
|  |  |  |  | 80 | 87 | 93 | 101 | 103 | 297 | 300 | 305 | 323 | 426 | 564 | 568 |
| EF418569 | Lion | FPV | Portugal | K | M | K | T | V | S | A | D | D | N | N | A |
| AB054227 | Tiger | TPV | Japan | K | M | K | T | V | S | A | D | D | N | N | A |
| AY955826 | Tiger | TPV | China | K | M | K | I | V | S | A | D | D | N | N | A |
| DQ099430 | Tiger | TPV | China | K | M | K | T | V | S | A | D | D | N | N | A |
| EF418568 | Tiger | TPV | Portugal | K | M | K | T | V | S | A | D | D | N | N | A |
| EF988660 | Tiger | TPV | China | K | M | K | T | V | S | A | D | D | N | N | A |
| EU252146 | Tiger | TPV | Korea | K | M | K | T | V | S | A | D | D | N | N | A |
| EU697386 | Tiger | TPV | China | K | M | K | T | V | S | A | D | D | N | N | A |
| EU697387 | Tiger | TPV | China | K | M | K | T | V | S | A | D | D | N | N | A |
| EU498692 | Tiger | TPV | India | K | M | K | T | V | S | A | D | D | N | N | A |
| EU697383 | Tiger | TPV | China | K | M | K | T | V | S | A | D | D | N | N | A |
| EU697384 | Tiger | TPV | China | K | M | K | T | V | S | A | D | D | N | N | A |
| FJ405225 | Tiger | TPV | China | K | M | K | T | V | S | A | D | D | N | N | A |
| KX685354 | Tiger | TPV | China | K | M | K | T | V | S | A | D | D | N | N | A |
| KX900570 | Tiger | TPV | China | K | M | K | T | V | S | A | D | D | N | N | A |
| MN908257 | Tiger | TPV | China | K | M | K | T | V | S | A | D | D | N | N | A |
| M38246 | Cat | FPV | China | K | M | K | I | V | S | A | D | D | N | N | A |
| EU221279 | Cat | FPV | Portugal | K | M | K | T | V | S | A | D | D | N | N | A |
| EU498680 | Cat | FPV | Italy | K | M | K | I | V | S | A | D | D | N | N | A |
| EU498681 | Wild cat | FPV | Italy | K | M | K | T | V | S | A | D | D | N | N | A |
| KP280068 | Cat | FPV | China | K | M | K | T | V | S | A | D | D | N | N | A |
| MN400978 | Cat | FPV | South Korea | K | M | K | T | V | S | A | D | D | N | N | A |
| MN400979 | Cat | FPV | South Korea | K | M | K | T | V | S | A | D | D | N | N | A |
| EU698028 | Blue fox | BFPV | China | K | M | K | I | V | S | P | D | D | N | N | A |
| U22185 | Blue fox | BFPV | USA | K | M | K | T | V | S | V | D | D | N | N | A |
| JF422105 | Mongoose | BFPV | PRT | K | M | K | T | V | S | A | D | D | N | N | A |
| D00765 | Mink | MEV | Japan | K | M | K | T | V | S | A | D | D | N | N | A |
| FJ712218 | Mink | MEV | China | K | M | K | T | V | S | I | D | D | K | N | A |
| FJ712219 | Mink | MEV | China | K | M | K | T | V | S | V | D | D | N | N | A |
| M24001 | Mink | MEV | USA | K | M | K | T | V | S | V | D | D | N | N | A |
| MW650832 | Dog | CPV-2 | USA | R | M | N | I | A | S | A | D | N | N | S | G |
| KC196108 | Dog | CPV-2c | China | R | L | N | T | A | A | G | Y | N | E | S | G |
| M24003 | Cat | CPV-2a | Crandell | R | L | N | T | A | A | G | Y | N | E | S | G |
| AF306444 | Wolf | CPV-2b | Italy | R | L | N | T | A | A | G | Y | N | D | S | G |
| M38245 | Canine | CPV-2 | USA | R | M | N | I | A | S | A | D | N | N | S | G |
| KF676668 | Canine | New CPV-2a | China | R | L | N | T | A | A | D | H | N | N | S | G |
| KC881278 | Canine | New CPV-2b | China | R | L | N | T | A | A | G | Y | N | D | S | G |
